# Interpreting the Terahertz Spectrum of Complex Materials: The Unique Contribution of the Bayesian Analysis

**DOI:** 10.3390/ma12182914

**Published:** 2019-09-09

**Authors:** Alessio De Francesco, Luisa Scaccia, Marco Maccarini, Ferdinando Formisano, Eleonora Guarini, Ubaldo Bafile, Alessandro Cunsolo

**Affiliations:** 1 Consiglio Nazionale delle Ricerche, Istituto Officina dei Materiali, Operative Group in Grenoble (OGG), c/o Institut Laue Langevin, 38042 Grenoble, France; 2 Dipartimento di Economia e Diritto, Università di Macerata, Via Crescimbeni 20, 62100 Macerata, Italy; 3 Laboratoire TIMC/IMAG UMR CNRS 5525 Grenoble, Université Grenoble-Alpes, 38000 Grenoble, France; 4 Dipartimento di Fisica e Astronomia, Università di Firenze, via G. Sansone 1, I-50019 Sesto Fiorentino, Italy; 5 Consiglio Nazionale delle Ricerche, Istituto di Fisica Applicata “Nello Carrara”, via Madonna del Piano 10, I-50019 Sesto Fiorentino, Italy; 6 Brookhaven National Laboratory-National Synchrotron Light Source-NSLS II, P.O. Box 5000, Upton, NY 11973, USA

**Keywords:** terahertz spectroscopy, Bayesian analysis, model choice, liquids dynamics, inelastic neutron scattering, inelastic X-ray scattering

## Abstract

In the last few decades, experimental studies of the terahertz spectrum of density fluctuations have considerably improved our knowledge of the mesoscopic dynamics of disordered materials, which also have imposed new demands on the data modelling and interpretation. Indeed, lineshape analyses are no longer limited to the phenomenological observation of inelastic features, as in the pioneering stage of Neutron or X-ray spectroscopy, rather aiming at the extraction from their shape of physically relevant quantities, as sound velocity and damping, relaxation times, or other transport coefficients. In this effort, researchers need to face both inherent and practical obstacles, respectively stemming from the highly damped nature of terahertz modes and the limited energy resolution, accessible kinematic region and statistical accuracy of the typical experimental outcome. To properly address these challenges, a global reconsideration of the lineshape modelling and the enforcement of evidence-based probabilistic inference is becoming crucial. Particularly compelling is the possibility of implementing Bayesian inference methods, which we illustrated here through an in-depth discussion of some results recently obtained in the analysis of Neutron and X-ray scattering results.

## 1. Introduction

Although in the last few decades the collective terahertz dynamics of disordered materials has been the focus of an intensive experimental, computational, and theoretical scrutiny, it still presents many obscure aspects. Experimental insight on this area could be gained from spectroscopic measurements at different values of exchanged energy ℏω and momentum ℏQ, with *ℏ* being the reduced Planck constant. Specifically, by proper tuning of the (Q,ω) variables, the dynamic response of the system can be probed as an average over different space-time domains. At extremely low (Q,ω)s, such an average involves distances far exceeding adjacent atom separations and time-lapses including many microscopic events, such as, e.g., rapid bounces of single atoms inside their first-neighbor shells. Under these circumstances, the hydrodynamic theory for continuous media provides a consistent description of the spectrum of density fluctuations [1] and it leads to predict the emergence of three long-living, or quasi-conserved, spectral modes stemming from the conservation laws of mass, momentum and energy. Upon increasing the exchanged momentum *Q*, these collective modes become more damped while involving smaller volumes (∝ $Q^{-3}$) and, consequently, fewer atoms. This trend would suggest that the dynamic response of the fluid loses any collective character when distances and times approach from the above adjacent atoms’ separations and in-shell atomic oscillation periods, respectively. Conversely, numerous experimental and computational results demonstrated that condensed materials defy this expectation. In fact, atoms in these systems are so tightly packed and interactions so short-ranged and rapid that even at mesoscopic scales the system response in the picosecond range is averaged over a significant number of elementary dynamic events. A somehow counter-intuitive consequence of this “close-packing” scenario is that an extended hydrodynamic-like spectral behavior persists down to mesoscopic scales and the very notion of “collective mode” preserves therein a definite physical meaning [2,3]. Although the endurance of a generalized hydrodynamic behaviour down to these scales is nowadays very well-assessed both experimentally and computationally, the lack of rigorous quantitative predictions for the shape of the spectrum as well as the highly damped character of spectral modes in this regime often prevents a rigorous modelling of the lineshape. Furthermore, the lineshape acquisition and subsequent interpretation are challenged by complementary resolution and kinematic limitation affecting the two experimental techniques in principle best suited to shed light on them: Inelastic X-ray (IXS [4]) and Neutron Scattering (INS [5]).

In the absence of a firm analytical prediction, the studies of both the ω- and the *Q*-evolution of the line-shape are mainly phenomenological in spirit: from one side, the ω-dependence is tackled by assuming a limited number of reasonable, yet arbitrary, *ansatz* on the scattering profile, entailing, for instance, the number of spectral modes, the coupling with different relaxation phenomena and so forth. On the other side, the *Q*-dependence of shape parameters is only determined heuristically by simply best-fitting such a model to the spectra measured at various *Q* values. Such a best-fit procedure is achieved by a conventional chi-square minimization routine, whose outcome, unfortunately, might critically depend on the *ansatz* adopted, which ultimately cast doubts on the overall reliability of the modelling. The need for a global reconsideration of the lineshape approach is becoming even more urgent when considering the interpretative challenges imposed by new-generation terahertz experiments on soft matter samples, as hybrid metamaterials and nanostructures, whose dynamic complexity has manifold aspects. Namely:

(1) First, the hybrid, solid–liquid, character of these materials makes in principle the spectral shape a complex superposition of liquid-like and solid-like features not trivially sorted out in the modelling.
(2) In addition, the existence of a mesoscale organization, with typical size λ, originates a break in the system homogeneity, i.e., its translation invariance, which expectedly emerges for *Q* ≈ 2π/λ. From a theoretical perspective, this homogeneity breach can be tackled by adding further variables in the description of the spectrum, which ultimately translates into the emergence of additional spectral modes.
(3) Finally, the *Q*-dependence becomes, in turn, exceptionally complex and, in principle, characterized by multiple *Q*-regimes. For instance, it can be expected that, for *Q* > 2π/λ, collective excitations involving the nanostructure interiors acquire increasing relevance.

To sort out this puzzling interpretative scenario, a minimal requirement is to limit the inherent bias of the adopted modelling, while opting for an evidence-based analysis of experimental results. Regardless of the scheme adopted, a few fundamental questions ultimately need to be explicitly addressed as directly impacting the overall integrity of the performed analysis. These include:

(1) how various competitive models perform in describing and explaining experimental data; (2) how to cope with the risk of an over-parametrization, i.e., of an unreasonably large number of free parameters in the model; (3) how to circumvent common fitting routine shortcomings as the convergence to local, instead of global, chi-square minima, and, most importantly, (4) how to implement minimally invasive external constraints without losing information from our prior knowledge (if any) of the physical problem at hand, and (5) how to efficiently cope with the limited statistical accuracy of experimental results by enhancing the inferential power of data analysis [6].

All these points can be successfully addressed by employing Bayesian inferential methods [7,8]. When applied to the analysis of spectroscopic results, these methods can enable probabilistic hypothesis tests involving, for instance, either the number of inelastic modes most likely to appear in the spectrum or the number of relaxation phenomena coupling with it. In the inference procedure, these unknown numbers can be treated as parameters themselves. Even more interesting is the possibility of submitting to a hypothesis test not only the number of relaxations associated with the observed dynamics, but also the model which better describes the available data. A very instructive example, not discussed in this short review because it refers to a time scale different from the one tackled here, is provided in Ref. [9] where, not only the finite number of decay channels through which relaxes the time correlation function of a polymer coated gold nanoparticles water solution is estimated, but also the nature itself of such decay channels. Certainly, the choice of the model is among a limited class of possible ones and in this particular case between combinations of simple exponentials, stretched exponentials, mixtures of stretched and unstretched exponentials or a simply Kohlrausch–Williams–Watts function [10].

Bayesian methods have been already successfully exploited to extract accurate structure factor amplitudes in regions of a powder diffraction pattern where Bragg peaks are widely overlapping [11], or to determine how many excitation lines are present in an inelastic neutron or X-ray scattering spectrum [12,13]. Another case where a Bayesian approach can efficiently enable a probabilistic model choice is the determination of the number of diffusive processes affecting density correlation function either in frequency [14,15] or in the time domain [9].

In general, the use of a Bayesian inference analysis is recommended when experimental results are noisy, or the spectral features weak, substantially damped or loosely resolved by the measurement. This method addresses the problem of the modelling from a perspective alternative to the so-called frequentist approaches, which are based on the traditional notion that the frequency of a given outcome “converges” to its probability after an infinite number of experimental attempts. The circumstance that routine experimental activity primarily consists of single events—as, e.g., a single spectral acquisition—rather than a repetitive chain of independent measurements, makes probabilistic inference uniquely valuable as support of data interpretation.

**Figure 1:**
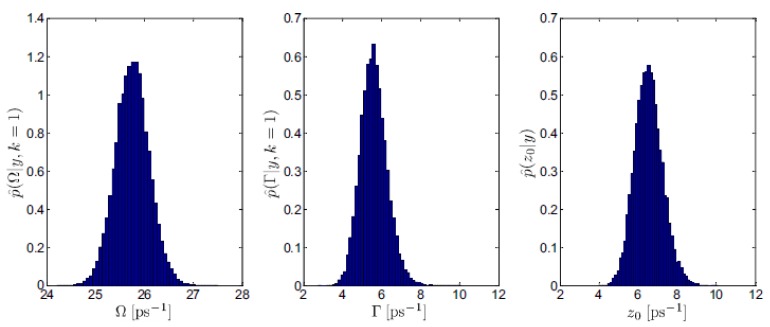
An example of the posteriors obtained from the Bayesian analysis of the INS spectra collected on liquid Au at *Q* = 10 nm${}^{-1}$. Reproduced from Ref. [12]. Copyright (2016) of American Physical Society.

Indeed, the main aspect distinguishing Bayesian approaches from the frequentist ones, is that the former ones deliver a probability distribution of each model parameter (Figure 1 shows an example of the posterior distributions for the model parameters employed in the analysis of INS data on liquid gold).

Furthermore, Bayesian estimates of model parameters are typically implemented through averages rather than optimization procedures; therefore, they cannot be misled by the proximity of fictitious (local) optima. Most importantly, unnecessarily complex models are penalized by the assignment of low weight (low likelihood) in the average [12], which naturally incorporates the Occam’s razor principle in the method [16]. According to this principle, among competitive hypotheses providing a satisfactory account of experimental evidence, the simplest one is to be privileged.

## 2. The Bayesian Method

The Bayesian inference method we discuss here was first successfully tested on Brillouin neutron scattering data of a crystal of UFe${}_{2}$ already published [17,18] and successively applied to INS measurements on liquid gold, as thoroughly discussed in Ref. [12], as well as IXS [19] and neutron Spin Echo [9] measurements on an aqueous suspension of Au nanoparticles.

Furthermore, possible alternative applications are reviewed in Ref. [13]. Thus, for instance, one can start from writing the dynamical structure factor as the sum of a finite number of excitations in the form:

(1) $$\begin{array}{cc} S(Q,E)= & A_{e}\left(Q\right)\delta \left(E\right)+\left[n\left(E\right)+1\right]\frac{E}{k_{B}T}\{L_{A_{0},z_{0}}\left(Q,E\right)+\\ & \sum_{j=1}^{k}\frac{2}{\pi }A_{j}\left(Q\right)DHO_{j}\left(Q,E\right)\},\; \end{array}$$

where E=ℏω is the energy transferred from the probe particle to the target sample, δ(E) the Dirac delta function describing the elastic response of the system defined by an intensity factor $A_{e}\left(Q\right)$, $n\left(E\right)={\left(e^{E/k_{B}T}-1\right)}^{-1}$ is the Bose thermal factor expressing the detailed balance condition, and the term in curly brackets is the sum of a Lorentzian central contribution—having half width at half maximum, $z_{0}$ and amplitude $A_{0}$—accounting for a quasielastic mode—and *k* inelastic contributions, accounted for by Damped Harmonic Oscillator ($DHO_{j}\left(Q,E\right)$) terms having amplitudes $A_{j}\left(Q\right)$:

(2) $$\begin{array}{c} DHO_{j}\left(Q,E\right)=\frac{\Omega_{j}^{2}\left(Q\right)*\Gamma_{j}\left(Q\right)}{{\left(E^{2}-\Omega_{j}^{2}\left(Q\right)\right)}^{2}+4{\left[E\Gamma_{j}\left(Q\right)\right]}^{2}}, \end{array}$$

where $\Omega_{j}\left(Q\right)$ and $\Gamma_{j}\left(Q\right)$ are the undamped energies and the damping coefficients of the DHO excitation. Notice that the number *k* of $DHO_{j}\left(Q,E\right)$ excitations likely to appear in the spectrum and their shape coefficients are equally treated as adjustable parameters. To provide an accurate approximation of the measured spectrum, the model function in Equation (1) must be convoluted with the instrument resolution function R(Q,E) and the result is assumed to sit on the spectral background. Explicitly:

(3) $$\tilde{S}\left(Q,E\right)=R\left(Q,E\right)⊗S\left(Q,E\right)+B\left(E\right),$$

where B(E) is a mildy *E*-dependent background intensity. The foundation of the used inferential method is the Bayes theorem:

(4) P(Θ|y,I)∝P(y|Θ,I)×P(Θ|I).

Here, the symbol “|” means “conditional on”, Θ is a vector having the model parameters as components, the vectors *y* and *I* respectively symbolize the spectral counts and any available prior knowledge on the problem. In the following, as generally done, the conditioning on *I* is omitted, even though implied, in order to lighten the notation. The first term on the right-hand side of Equation (4) is the likelihood function, representing the probability of the observed experimental data, conditional on both parameters and prior knowledge. The second term is the prior distribution of the model parameters based upon the information available before data collection. The left-hand side of Equation (4) is the joint posterior distribution of the parameters that is the probability distribution to be assigned to parameters once data have been collected and conditional on the stated prior knowledge. Let us start from defining the likelihood function for the data at hand. In general, experimental data are affected by additive random errors to be represented in the form:

$$y_{i}=\tilde{S}\left(Q,E_{i}\right)+\epsilon_{i},\;for\;i=1,\ldots ,n,$$

where $\tilde{S}\left(Q,E_{i}\right)$ is the model of the spectral profile in Equation (3), $E_{i}$ is the energy position of the *i*th experimental observation and *n* is the number of experimental data points. We assume the random errors $\epsilon_{i}$ to be independently and identically distributed according to a normal distribution $N(0,\nu \sigma_{i}^{2}),$ where $\sigma_{i}$ is the experimental error corresponding to the *i*th observation and ν is a proportionality constant. The likelihood of the data then reads as:

(5) $$\begin{array}{cc} P\left(y|\Theta \right)=\prod_{i=1}^{n}\frac{1}{\sqrt{2\pi \nu \sigma_{i}^{2}}}\exp\left(-\frac{{\left[y_{i}-\tilde{S}\left(Q,E_{i}\right)\right]}^{2}}{2\nu \sigma_{i}^{2}}\right)= & \\ =K\exp\left(-\sum_{i=1}^{n}\frac{{\left[y_{i}-\tilde{S}\left(Q,E_{i}\right)\right]}^{2}}{2\nu \sigma_{i}^{2}}\right). \end{array}$$

Although any available a priori information on the problem should be included, whenever this is not available, uninformative (uniform) priors can still be used. This option leads to retrieve the same fitting results as the conventional least-squares approach, with the additional estimate of related probability distributions and probabilistic support for the model chosen. Notice that the equivalence between Bayesian and frequentist results remains valid asymptotically, i.e., for an infinitely large number of experimental points, even in the presence of informative priors.

After specifying likelihood and priors for each parameter, the Bayes theorem in Equation (4) enables extracting the posterior probability distribution for each of these parameters up to a normalization constant. However, the actual computation of this constant would require an analytically prohibitive high-dimensional integration, generally circumvented by using Markov chain Monte Carlo methods (MCMC), which allow for simulating the joint posterior distribution of the parameters. Details about these methods can be found in Ref. [12] and references therein. Notice that the hyperspace of model parameters has varying dimension since the latter depends on the variable parameter *k*. For this reason, the MCMC algorithm is incremented by a reversible jump (RJ) step [20]. The resulting RJ-MCMC algorithm not only can span the parameter space but also easily switch between spaces of different dimension or, equivalently, jump between models containing a different number of excitations. After an initial burn-in period enabling the convergence of the Markov chain, each algorithm sweep updates all the parameters of the model, including *k*, in turn, drawing a new parameter value from its posterior distribution, conditional on the data and the values of all the other parameters. Thus, at each sweep *m*, the algorithm produces a complete draw, $\Theta^{\left(m\right)}$ from the joint posterior distribution of the whole parameter set. The result obtained for m=1,…,M, can be used to estimate all the quantities of interest. For instance, the posterior distribution of the number of spectral components *k* can be estimated as the relative frequency with which each model was visited by the algorithm, namely:

(6) $$P\left(k=l|y\right)=\frac{\sum_{m=1}^{M}J\left(k^{\left(m\right)}=l\right)}{M}=\frac{M_{l}}{M},$$

where $M_{l}$ is the number of times the model with *l* components was visited, while the indicator function *J* is equal to 1 when its argument is true and 0, otherwise.

The model with the highest posterior probability, i.e., the one most frequently visited by the algorithm, is selected as the best model to represent the data. Conditional on this best model, let us say the one with *l* spectral components, the parameters $A_{j}$, $\Omega_{j}$ and $\Gamma_{j}$, for j=1,…,l, can be estimated as the mean of their simulated marginal posterior distribution, i.e.:

(7) $${\hat{A}}_{j}=\frac{\sum_{m:k^{\left(m\right)}=l}A_{j}^{\left(m\right)}}{M_{l}},$$

(8) $${\hat{\Omega }}_{j}=\frac{\sum_{m:k^{\left(m\right)}=l}\Omega_{j}^{\left(m\right)}}{M_{l}},$$

and

(9) $${\hat{\Gamma }}_{j}=\frac{\sum_{m:k^{\left(m\right)}=l}\Gamma_{j}^{\left(m\right)}}{M_{l}},$$

where the sum index $m:k^{\left(m\right)}=l$ means that the sum is performed over all sweeps *m* such that $k^{\left(m\right)}=l$ and we dropped the *Q* dependence in the model parameters. All the other parameters do not depend on the number of spectral components and can be estimated as the mean of their simulated marginal posterior distributions, averaging over all the sweeps of the algorithm. Notice that, being the simulated marginal posterior distribution of a parameter not necessarily symmetric, other estimators can be more appropriate than the arithmetic mean, as, e.g., the simulated posterior mode. Given this succinct introduction to the used procedure, here we review a couple of recent works discussing a Bayesian analysis of Inelastic Scattering measurement as performed with either X-ray or neutron spectroscopy probes.

## 3. Discussion of Results

As mentioned in the introductory section, the detection of clear inelastic peaks at mesoscopic scales is hampered by both instrumental and inherent physical difficulties: the former mainly relate to the large width and, for IXS, to the slowly decaying tails of the resolution profile, which often prevent a clear detection of low-frequency spectral features; conversely, the second are intrinsic difficulties due to the highly damped nature of collective modes in disordered systems. As a consequence, when dealing with liquids and amorphous systems, the spectra measured by IXS and INS usually look somewhat unstructured with inelastic features often barely discernible at first sight. A partial exception is liquid metals whose spectral profiles bear evidence of distinct features up to *Q* values as high as about 2/3 of the position of the main sharp diffraction peak [21]. In the past, this peculiarity was ascribed to the soft repulsive portion of the interatomic potential [22] as opposite to the harsh, hard-sphere-like, core interactions of atoms in noble gases, whose neutron spectrum is indeed essentially featureless for *Q* larger than a few nm${}^{-1}$ [23,24]. In addition, in some liquid metals, the spectral contribution from thermal fluctuations is usually weak, i.e., a specific heats ratio γ is close to one, which sensibly reduces the intensity of the central, Rayleigh peak of the spectrum, thus enhancing the visibility of inelastic features. Another inherent reason of simplicity is that, in liquid metals, viscoelastic effects, such as the well known *Q*-increase of sound velocity, are relatively small, which, in turn, has been associated with the especially soft nature of atomic interactions [3]. Finally, inelastic excitations in liquid metals have been often ascribed to longitudinal acoustic modes. This point is important and very much so considering that there is an increasing number of IXS and INS work documenting the presence of non-longitudinal modes in the terahertz spectrum of density fluctuations. The assignment of shear, or transverse, polarisation to a mode might appear surprising, as density fluctuations couple primarily with longitudinal movements only. Indeed, the coupling of density fluctuations with transverse waves can occur through some entanglement between longitudinal and transverse acoustic modes, customarily referred to as longitudinal-transverse coupling (LTC) [25].

Evidence of an LTC is being reported for a growing number of systems. Initially, this effect seemed to mostly occur in tetrahedrally networked fluids such as water [25,26], SiO${}_{2}$[27,28], GeO${}_{2}$[29] and GeSe${}_{2}$ [30], which suggested ascribing this effect to the highly directional and intrinsically open character of the tetrahedral arrangement. However, it seems now well agreed that LTC is not a prerogative of tetrahedral arrangements as it was reported in a computer simulation of a non-tetrahedrally networked system as glassy glycerol [31] and liquid methanol [32,33]. Even more surprisingly, an LTC effect was observed in tightly packed and non-associated systems, as liquid metals. This is, for instance, the case of a seminal work on liquid lead [34] and more liquid Ga [35,36], Na [37] and Zinc [38].

**Figure 2:**
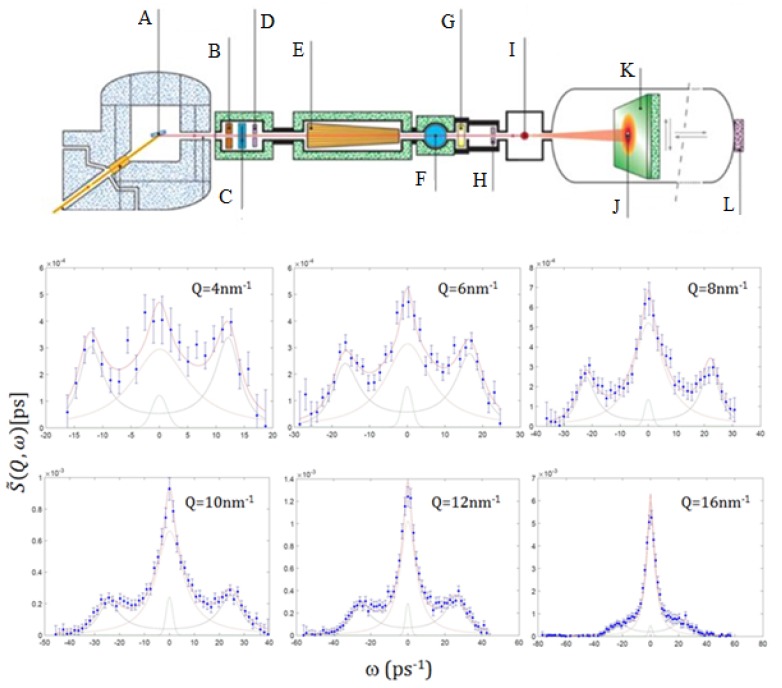
Top panel: A schematic layout of BRISP, the NBS instrument used to collect the spectra reported in the figure, whose main components are (from left to right) (**A**) monochromator, (**B**) shutter, (**C**) background chopper, (**D**) first diaphragm (**E**) 2D collimator, (**E**) Fermi chopper, (**G**) monitor, (**H**) second diaphragm, (**I**) sample, (**J**) first beam stop, (**K**) position sensitive detector, (**L**) second beam stop. Bottom panels: Dynamic structure factor of liquid gold measured on BRISP at the *Q* values indicated in the panels. The experimental data (blue dots) are reported along with RJ-MCMC best-fit (red continuous line) and the individual spectral components for the most plausible model option, corresponding to *k* = 1. Adapted from Ref. [12]. Copyright (2016) of American Physical Society.

Given these grounds, a liquid metal would represent an ideal benchmark sample to test a Bayesian inference analysis of the spectral shape, and this motivated our Neutron Brillouin Scattering (NBS) study on liquid gold [12]. In Figure 2, representative NBS spectra collected on liquid Au are compared with their best fitting lineshape for the most plausible model option (*k* = 1), along with the individual, quasielastic and inelastic, spectral components. It can be readily appreciated that inelastic modes show up in the spectrum as well resolved side peaks, apparently becoming increasingly damped as *Q* increases, up to turn into loosely resolved spectral shoulders at *Q* = 16 nm${}^{-1}$.

**Figure 3:**
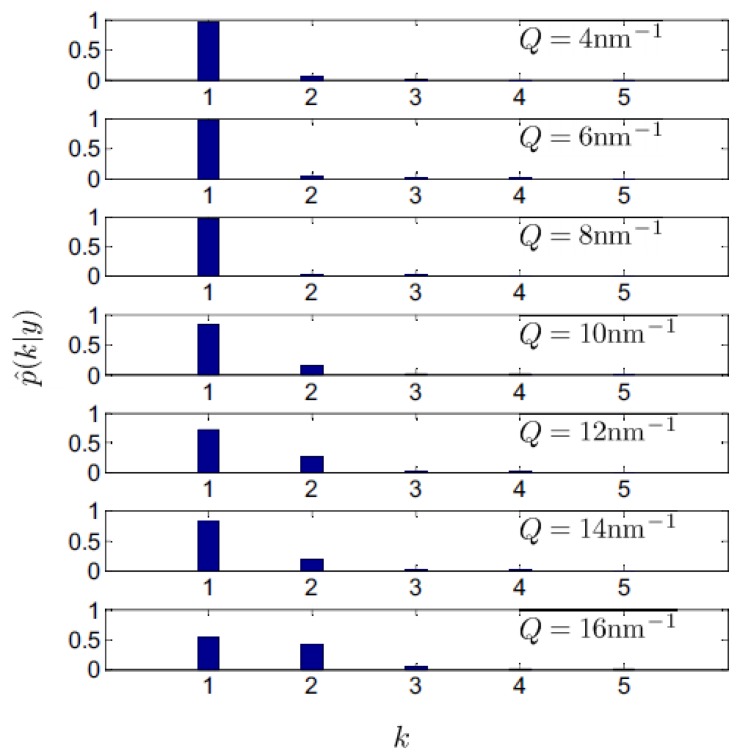
Posterior probability for the number of modes *k* for the various *Q* values probed in the INS measurement on liquid gold. Ref. [12]. Copyright (2016) of American Physical Society.

In Figure 3, we report the posterior probability of the number of spectral modes, *k*, contributing to the scattering profile measured at various *Q*’s. Due to our agnostic a priori guess on the actual value of *k*, we used a uniform prior distribution for this parameter, which made the inference on its most plausible value entirely grounded on experimental evidence. The plot in Figure 3 shows that probability of the most plausible model option (*k* = 1) becomes lower as *Q* increases, this trend being paralleled by the increasing probability of the *k* = 2 alternative model option.

**Figure 4:**
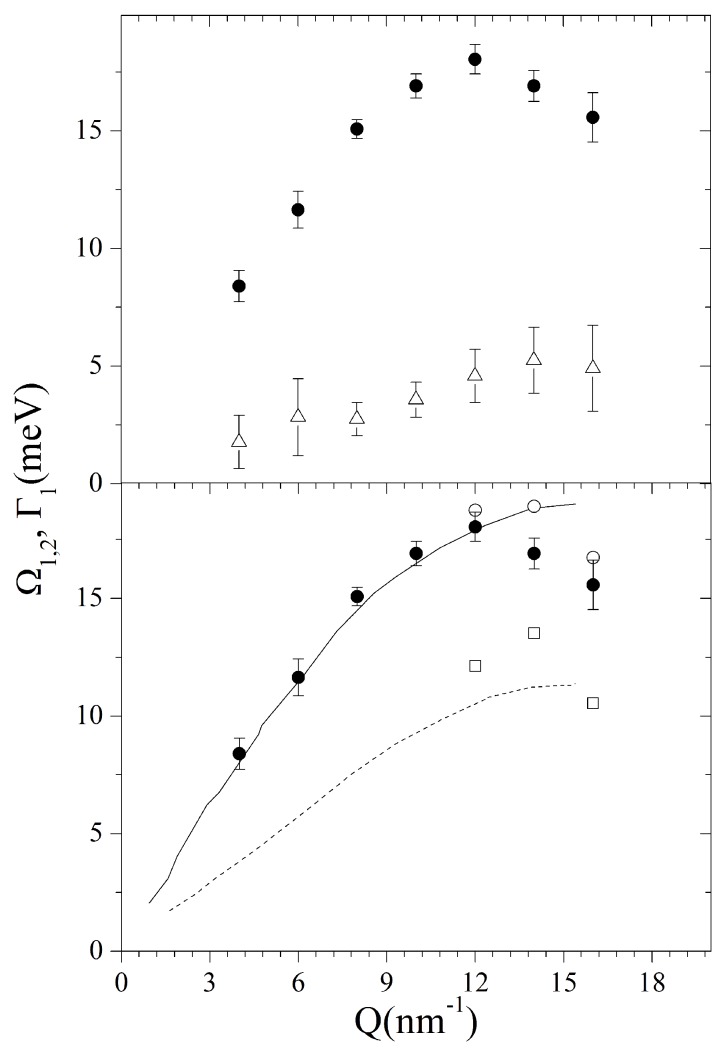
**Top panel**: Frequency (dots) and damping (triangles) of the acoustic modes of liquid gold as derived through through a DHO-based model and the RJ-MCMC algorithm. Redrawn from Ref. [12]. Copyright (2016) of American Physical Society. **Bottom panel**: The only mode frequency in liquid gold corresponding to the k=1 option (see text) as in the top panel (black filled circles) is plotted together with the two frequency dispersions would be obtained at the higher *Q* values always in liquid gold, if the k=2 option would be chosen (empty squares and circles) and the acoustic phonon branches measured in solid Au (dashed and solid black lines).

The maxima of the $\Omega_{1}$ posterior distributions corresponding to the most plausible, *k* = 1, model option enabled us to build up the dispersion curves plotted in Figure 4.

From the top plot of such a figure, it clearly appears that the undamped frequency $\Omega_{1}$ largely exceeds its damping; this trend confirms the nearly ubiquitous observation of long-living terahertz collective excitations in liquid metals (see the review in Ref. [21]), even though the relative damping appears to enhance systematically at the highest *Q*’s. In addition, the sound dispersion exhibits a linear trend up to moderate *Q*’s, only slightly exceeding the hydrodynamic dispersion, $c_{s}Q$ with $c_{s}$ = 2658 m/s, being the sound velocity of liquid gold [39]. Upon increasing *Q*, $\Omega_{1}$ values bend downwards due to the onset of a destructive interference between the acoustic mode and the first neighboring atomic shell. In the bottom panel of the same figure, best-fit values of $\Omega_{1}$ corresponding to the *k* = 1 model option are compared with the values of $\Omega_{1,2}$ for the k=2 option. Furthermore, the acoustic phonon branches measured in crystalline Au [40] along the [100] direction are also included for reference. The favorable comparison of the sound dispersions in the two samples might support the assignment of the alleged second feature in the liquid to a transverse acoustic mode. Interestingly, the onset of this additional mode at high *Q* is not inconsistent with the behavior of shear modes in the spectrum of other amorphous materials, including liquid water [41]. However, the hardly comparable temperatures and structures of the samples under comparison warn us against hasty conclusions, leaving intact the doubt that inference on *k* might have been misled by the broader and less resolved inelastic features in the highest *Q* spectra. Furthermore, the inference is further challenged by kinematic limitations, which prevent an adequate coverage of remote spectral tails.

In any case, the Bayesian approach should never betray its natural mission, which is not surrogating researchers, but rather assisting them with a robust probabilistic ground, without limiting their ultimate authority and responsibility in interpreting the experimental outcome. Our previous *ab initio* computation of Au spectra of liquid Au [42] showed that a viscoelastic modeling provides a more accurate approximation of the S(Q,ω), especially in the low-frequency portion dominated by the “relaxation”, or Mountain, mode [43]. It can be concluded that the low-frequency spectral intensity “excess” (either measured or computed) can be alternatively “filled” by inserting in the model either a second excitation, as discussed here, or a quasielastic relaxation mode, as suggested by our previous *ab initio* analysis. This ambiguity originates two crucial questions: (1) Given the sets of experimental and computational results can a plausibility test of either hypothesis be envisaged? (2) Does a sharp distinction between them make any physical sense at all? The first question defines the case for a possible hypothesis test based on Bayesian inference methods; the second question instead eludes the domain of evidence-based interpretations. Our opinion is that a clearcut distinction might have a limited physical significance due to the high relative damping of the alleged low-frequency spectral mode which could hinder any firm distinction between its propagating or relaxational nature. Again, in the spirit of the Occam’s razor, we would tend to privilege the viscoelastic hypothesis for describing S(Q,ω), if nothing else, at least because it entails a lower number of free parameters. Given the information at hand, any conclusion on the physical problem described thus far would be a bold speculation not strictly authorized by an unbiased observation of results. On a general ground, it appears that the physical origin of largely damped inelastic features often represents a grey area in which competing guesses on the lineshape sometimes can hardly be disproved or corroborated. Instrumental factors such as resolution or kinematic limitations, or the poor statistical accuracy of the beam counting, make the scenario even foggier further increasing the risk of over-interpretations and unjustified claims. This being said, additional insight on this hypothetical second mode can be shed by a dedicated molecular simulation on a model system representative of liquid Au, directly accessing transverse and longitudinal current spectra. This was indeed done by direct calculation of the transverse current-current spectrum and also by resorting to the important information on collective modes that is unavoidably contained also in single-particle correlation functions like the self dynamic structure factor and the spectrum of the velocity autocorrelation function [44].

**Figure 5:**
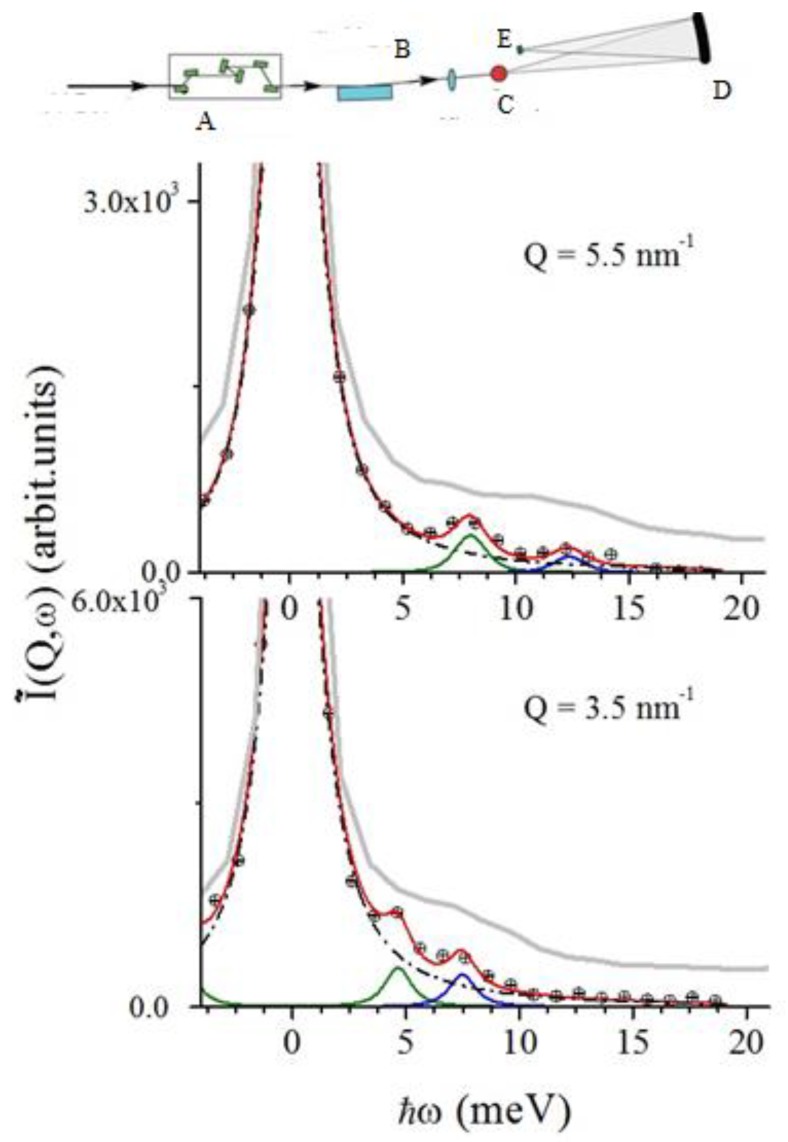
**Top panel**: the IXS instrument used to perform the measurements reported, whose main components are: (**A**) in-line monochromator, (**B**) beam focusing unit, (**C**) sample (**D**), spherical analysers (nine simultaneously working in a single scan), (**E**) detector. **Bottom panel**: IXS spectra (open circles) of the Au NP suspension measured at few representative *Q* values. The best fit lineshapes by the RJ-MCMC algorithm are also reported as red solid lines along with the low (green solid line) and high-frequency (blue solid line) inelastic excitations as well as the quasielastic Lorentzian component (black dashed-dotted line). Finally, the spectra measured on pure water in a previous IXS work [54] at close *Q* values (*Q* = 4 nm${}^{-1}$ and 6 nm${}^{-1}$ in the lower and upper panel respectively) are also reported as a thick grey line after a re-scaling to the same maximum of the colloidal suspension spectrum. Error bars on the measured spectral shapes are within the symbol size. Reprinted with permission from Ref. [19]. Copyright (2018) American Chemical Society.

Let us now consider a terahertz spectral shape in principle more complex as the one of a hybrid, solid-liquid, material, as a colloidal suspension. Specifically, here we deal with the case of an inelastic X-ray scattering (IXS) measurement on a monodisperse aqueous suspension of Au nanospheres with a diameter *d* = 50 nm. Expectedly, the spectrum of this sample contains both liquid-like and solid-like acoustic modes, yet only for wavelengths 2π/Q≤d, since phonon modes of longer wavelength are not allowed to propagate inside the nanoparticles (NP) interior. The rather dilute nature of the suspension considered, about 1% volume NP concentration in water, might suggest that its spectrum deviates only slightly from the one of the pure liquid substrate, water, thoroughly studied in the past by both IXS [26,45,46,47,48,49] and INS [50,51,52,53]. Figure 5 proves that this expectation misrepresented the spectral shape actually measured, which substantially differs from the one of pure water, also included in the plot for reference. To ease the comparison, the latter profile was rescaled to the maximum of the NP suspension spectrum and slightly offset vertically to avoid tail superposition. It can be readily noticed that the broad side shoulders in the spectrum of pure water, arising from a propagating collective mode, are hardly visible in the NP spectrum, where they are replaced by a sharp, yet weak, doublet. The presence of these additional peaks emerges perhaps more clearly using, as a guide to the eye, the best fitting lineshape corresponding to the most plausible model option inferred with the RJ-MCMC algorithm, which in this case was the one corresponding to k=2. Let us discuss here two important aspects of this result.

**Figure 6:**
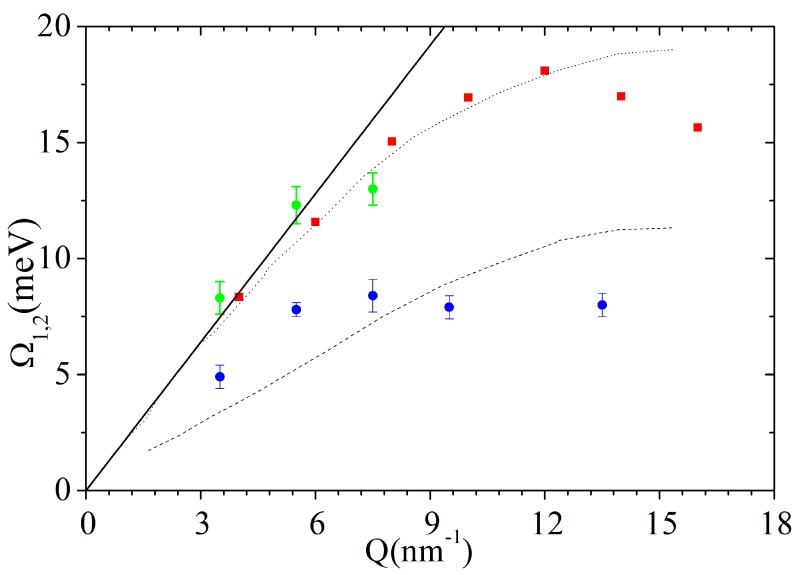
The dispersion curves of the high ($\Omega_{2}$) and low ($\Omega_{1}$) frequency inelastic modes of the Au NP suspension in water are reported as green and blue dots, respectively, as determined from the best fit provided by the RJ-MCMC Bayesian inference method described in the text. In addition, the acoustic phonon branches measured in crystal along the Au [100] direction (dash and dots black lines) and the sound dispersion of liquid Au corresponding to the *k* =1 hypothesis are shown as estimated in Ref. [12] (red squares). The plot also includes, as a solid line, the linear hydrodynamic dispersion obtained with the ambient *T* sound velocity (3240 m/s). Adapted with permission from Ref. [19]. Copyright (2018) American Chemical Society.

Although the inelastic peaks in the spectrum from the suspension are sharp and easily discernible, their shape is mapped by no more than one to two spectral points, clearly outstanding from the spectral wings by an amount larger than average count fluctuations. Remarkably, these spectral features are so narrow that their shape could only be consistently described by replacing the DHO excitations in the model with corresponding $\delta (\omega \pm \omega_{s})$ pairs. To some extent, this last point stresses even more the novelty of this result, as the Bayesian inference led to exclude at all the presence of any excitation with finite damping, as those instead dominating the inelastic portion of the spectrum of pure water, and, more in general, most spectra from amorphous materials. Indeed, both the sharp nature of these peaks and their *Q*-dispersive shift hint at the assignment to acoustic phonon modes propagating in the Au NP interior. A more informed conclusion can be reached by looking at the dispersion curves reported in Figure 6, which are therein compared with the dispersion branches in liquid and solid Au, already reported in Figure 4, along with the hydrodynamic linear dispersion evaluated using the ambient *T* sound velocity, $c_{s}$= 3240 m/s. The comparison shows that the values of $\Omega_{2}$ are fairly consistent with the frequency of the longitudinal sound mode of liquid bulk gold, both essentially merging into the linear dispersion upon approaching the low *Q* quasi-macroscopic limit. In addition, $\Omega_{1}$ values exhibit some agreement with the transverse phonon branch of crystalline gold. These findings summarize the main conclusions of the performed Bayesian inferential analysis: the only excitations appearing in this dilute aqueous colloidal suspension are the acoustic phonons propagating inside the NP interior, while no signature can be found of the collective modes dominating the inelastic tails of pure water spectrum.

**Figure 7:**
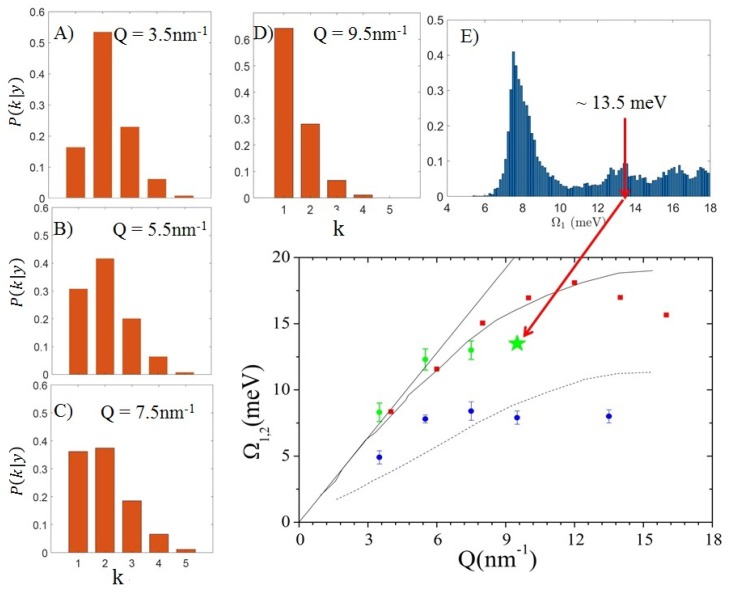
(**A**–**C**): posterior probability for the number of inelastic modes k for the IXS spectra collected on the NP suspension up to *Q* = 7.5 nm${}^{-1}$; the same probability for *Q* = 9.5 nm${}^{-1}$ is also reported in (**D**), while the posterior distribution $P(\Omega_{1}|y)$ for the excitation frequency corresponding to the most plausible model option at this *Q* value (*k* = 1) is reported in (**E**). Finally, the energy derived from the minor mode of this distribution is inserted as a star in the plot of Figure 6 (see arrow). Adapted with permission from Ref. [19]. Copyright (2018) American Chemical Society.

Let us now discuss the posterior probability of *k*. The values determined for the three lowest *Q* values are reported in Panels A÷C of Figure 7, from which it appears that the plausibility of the *k* = 2 option decreases upon *Q*-increase and finally when Q>7.5 nm${}^{-1}$ the most visited model becomes the one with k=1. Perhaps even more interesting is the case of the spectrum collected at Q=9.5 nm${}^{-1}$ for which fitting results are illustrated in Panels D÷F of the same figure. In particular, Panels D and E respectively display the *k* posterior probability and the corresponding posterior distribution of $\Omega_{1}$. It can be readily noticed that, although the latter distribution refers to the (most plausible) *k* = 1 model option, it has a multimodal profile characterized by a dominant peak at about 8 meV, consistent with the transverse acoustic phonon branch of Au, but also a weaker maximum at about 13.5 meV; this energy fits fairly well into the longitudinal phonon branch of Au, as it appears in Panel F which compares such an energy (as a star) to the dispersion curves of Figure 6. The one discussed above can be considered a nice example of the highly informative character of a Bayesian inference outcome, which not only identifies the most plausible value of a parameter corresponding to the preferred model option, but it gives clear indication on possible alternative values that such a parameter might attain, through the multimodal character of its posterior distribution. In summary, Panels A÷C suggest a systematic *Q*-increase of plausibility of the transverse phonon mode, which clearly reflects the corresponding relative enhancement of the low-frequency peak in the spectrum. At *Q* = 9.5 nm${}^{-1},$ the intensity of such a peak becomes so overwhelming that the single excitation (*k* = 1) hypothesis becomes the most plausible model option; however, this trend, in itself, does not exclude the presence in the measured spectrum of the longitudinal branch of the Au, as indicated by the onset of a second high-frequency mode in the $\Omega_{1}$ posterior distribution.

## 4. Conclusions

In conclusion, we have shown here that Bayesian methods can be successfully implemented in the analysis of terahertz spectroscopic measurements on disordered materials to achieve a robust evidence-based modelling of the spectrum and a probabilistic inference on spectral parameters. We hope that the full potentialities of these methods will be soon recognized by the scientific community, ultimately defining broadly accepted protocols in the data analysis. In the foreseeable future, we may anticipate that similar methods can be successfully used to perform joint analyses of multimodal experiments involving complementary terahertz techniques operating either in the frequency or in the time domain. The joint analysis of these independent results can be carried out with equipollent models linked via Fourier transform, using, for instance, a sum of complex exponentials to model experimental time correlation functions and the corresponding a sum of generalized Lorentzians [55], when dealing with measured spectra. In addition, we can envisage the use of more informative priors based, e.g., on the sum rule fulfillment, or the simultaneous fitting of multiple spectra aimed at testing competing hypothesis on their *Q* dependence, and so forth.

The INS and IXS measurements discussed in this short review have been carried out using, respectively, the BRISP spectrometer [56] at the high flux reactor neutron facility of the Institut Laue-Langevin in Grenoble, France and the HERIX spectrometer [57,58] at the Advanced Photon Source of Argonne National Laboratory in Lemont, IL, USA. Schematic layouts of these two spectrometers are reported in the top panels of Figure 2 and Figure 7, respectively, while further details on the measurements can be found in the original works, i.e., in References [12,19], respectively.
